# Impacts of varying day and night environmental conditions on cotton flowering, yield, and fiber quality

**DOI:** 10.3389/fpls.2025.1616982

**Published:** 2025-10-02

**Authors:** Naflath Thenveettil, Manoj Kumar Reddy Allam, Saseendran S. Anapalli, Krishna N. Reddy, Wei Gao, K. Raja Reddy

**Affiliations:** ^1^ Department of Plant and Soil Sciences, Mississippi State University, Starkville, MS, United States; ^2^ Crop Production Systems Research Unit, U.S. Department of Agriculture- Agricultural Research Service (USDA-ARS), Stoneville, MS, United States; ^3^ Department of Ecosystem Science and Sustainability, U.S. Department of Agriculture (USDA)- UV-B Monitoring and Research Program, Colorado State University, Fort Collins, CO, United States

**Keywords:** boll production, boll retention, fiber quality, ovules, pollen grains

## Abstract

**Introduction:**

Increases in the frequency of higher-than-optimum air temperatures can substantially reduce cotton production. Little is known about the influence of different combinations of day/nighttime temperature on cotton flowering and boll maturation under ambient and elevated CO_2_ conditions.

**Methods:**

This study examined the impacts of air temperature variations on the morphology of cotton flowers and seed yield under air CO_2_ concentrations at 425 ppm (ambient, aCO_2_) and elevated at 725 ppm (eCO_2_) in controlled Soil-Plant Atmospheric Research (SPAR) chambers. The four temperature conditions were: optimum (OT; 33/21°C, day/night), high temperature (HT; 36/24°C, day/night), high nighttime (OT+HNT; 33/24 °C, day/night), and high day/nighttime (HT+HNT; 36/28 °C, day/night).

**Results:**

Various reproductive and seed yield traits, as well as the phenology of the plants, differed significantly (p < 0.001) under the treatments. The boll maturation period significantly decreased in plants grown under HT+HNT, with only 39 days under aCO_2_ and 38 days under eCO_2_ compared to 47 days at OT. In the HT and OT+HNT conditions, the duration was 42 days at aCO_2_ and 46 days at eCO_2_, as opposed to 41 and 44 days, respectively, under aCO_2_. Furthermore, there was a significant reduction in the number of pollen grains per anther, 13% for OT+HNT, 24% for HT, and 39% for HT+HNT, relative to OT treatments. The seed cotton weight also showed a drastic decline, decreasing from 105 g plant^-1^ under OT to 90 g under OT+HNT, 47 g under HT, and 12 g plant^-1^ under HT+HNT conditions. In the HT+HNT environment, lint percentage and seed weight per plant were reduced by 26% and 86%, respectively, when compared to OT. The eCO_2_ did not alleviate the reductions in cotton yield caused by higher air temperature exposure.

**Discussion:**

This study highlights that high air temperature induces flower abscission and anther indehiscence, while diverting biomass allocation towards vegetative organs. The resulting source-sink imbalances between vegetative and reproductive structures resulted in significant reductions in seed and lint yield and growth patterns across CO_2_ and temperature environments. These findings provide insights into cotton management strategies under future environmental scenarios.

## 1 Introduction

Globally, cotton is the most widely produced and utilized natural fiber, with an annual economic value of around 600 billion USD (Khan et al., 2020). Among different cotton species, Upland cotton (*Gossypium hirsutum* L.) cultivars are the most widely cultivated, constituting 97% of the US production (USDA-ERS, 2024). In 2024, global cotton production reached 25 million tons, with the US contributing about 2.6 million tons from 11.2 million acres (MacDonald, 2024), accounting for 11% of total global production after China (24%), India (22%), and Brazil (13%) (USDA-FAS, 2024a). However, the US leads the cotton export market with an estimated export value of 5 billion USD (USDA-FAS, 2024b) holding a key role in the global cotton market. In 2024, the US cotton yield reduced by 7% compared to the previous year (USDA-NASS, 2024). This is attributed to the crop’s sensitivity to environmental cues like increased occurrences of heatwaves, prolonged drought, and other extreme weather events during crop growth and development (Amin et al., 2017). Increased carbon dioxide (CO_2_) emissions from fossil fuel consumption and land use changes have elevated the atmospheric CO_2_ concentration (|CO_2_|) to about 425 ppm in 2024 (Lindsey, 2024). The rate of increase in CO_2_ is currently around 2.5 ppm per year, and the consequent increase in absorbed heat energy in the atmosphere drives the rise in global temperatures.

According to NASA’s global air temperature monitoring and assessments, the average global annual temperature in 2024 was up by 1.28°C from its average during 1951 to 1980 (NASA, 2024). Notably, 2024 marked the warmest year on record since recordkeeping began in 1880. Since the 1970s, unusually hot summer days with both high day and nighttime temperatures have become more common over the last few decades in the United States (EPA, 2016). More specifically, the nighttime temperature in most locations is rising faster than the daytime temperature (Xia et al., 2014), contributing to a reduction in the diurnal temperature range (Easterling et al., 2000). In line with this trend, the global daily mean minimum temperature increased by 0.8°C per century, compared to a daily maximum temperature increase of 0.4 °C (NCEI, 2024). Plants exhibit modifications in physiological and growth characteristics under these changing temperature conditions (Impa et al., 2019). The rate of plant growth is dependent on the temperature, and the critical temperature for the crop (Hatfield and Prueger, 2015; Soni et al., 2025). Quantifying these modifications in plant growth and yield provides a rationale for exploring key traits that will help breed heat-stress-resilient cotton in the future.

The thermal kinetic window of cotton for optimum metabolic activity with maximum photosynthesis is at about 26-28°C (Burke et al., 1985). Higher than optimum temperatures promote growth and early maturation in cotton; however, they prevent plants from fully achieving their genetic potential (Reddy and Zhao, 2005; Saini et al., 2023). The response of plants to high-temperature conditions also depends on the growing conditions. Few field-based studies have reported that plant height, number of nodes, and internodal length decline under high-temperature conditions where the maximum growing temperature exceeds 40°C (Abro et al., 2021; Yousaf et al., 2023). At the same time, studies conducted under control conditions with optimum water and nutrients observed an increase in plant height and number of nodes with increasing growing temperatures (Reddy et al., 1992, 1998). However, all these studies agreed that high temperatures have an adverse effect on boll retention and cottonseed and lint yield. It can be explained by the imbalance between the source and sink, which adversely affects the yield of cotton (Nie et al., 2020; Qin et al., 2023). Similar to most crops, cotton is susceptible to heat stress during the reproductive phase (Salman et al., 2019; Farooq et al., 2023). It has been reported that high temperature stress deforms the reproductive organs, disrupting pollen maturation, germination, and ovule development, ultimately resulting in lower seed set and crop yield (Snider et al., 2009; Hatfield et al., 2018; Raja et al., 2019; Masoomi-Aladizgeh et al., 2021). The high night temperature (HNT) of 29-31°C is reported to have a disruptive effect on pollen development, leading to male sterility, mainly due to the limited supply of storage lipids and fatty acids as a result of increased respiration (Khan et al., 2020). These modifications have contributed to the increased square and flower abscission observed under high day and night conditions (Song et al., 2015; Loka and Oosterhuis, 2016; Bista et al., 2025).

High temperatures reduce the yield and yield-related parameters such as boll and seed number and lint yield in cotton (Abro et al., 2021; Yousaf et al., 2023). Research indicates that the impact of high-temperature stress becomes more severe when both day and night temperatures rise together, compared to an increase in either one alone (Zafar et al., 2021; Saini et al., 2023; Parkash et al., 2024). As high as a 50-60% reduction in seed and lint yield was observed under a high day and night temperature of 37/26 °C (Mishra et al., 2017). Similarly, the quality of cotton fiber is affected by the growing temperature conditions. A single seed generates around 10,000 to 20,000 fibers under ideal temperature conditions, which is around 26-28°C (Reddy et al., 1997). Temperatures above 35 °C can negatively impact the quality properties of the fiber, affecting its marketable value (Manan et al., 2022). A lower rate of photosynthesis, coupled with the disruption of enzymes involved in sucrose metabolism and cellulose synthesis, is the primary cause of reduced fiber quality under high-temperature conditions (Chen et al., 2017; Ding et al., 2021).

Although studies have been conducted to investigate the impact of high day and night temperatures on cotton yield and quality, a systematic study on the sole and combined effects of high day and nighttime temperatures on reproductive and yield-related traits under ambient and elevated CO_2_ concentrations has not been attempted before. The study aimed to (i) understand the boll production and retention over time under various combinations of high day and/or night temperatures and CO_2_, (ii) evaluate the effects of high day and/or night temperature stress on flower characteristics under different CO_2_ levels, and (iii) estimate the impact of these temperature and CO_2_ combinations on seed and lint yields and fiber quality, all under optimum water and nutrient conditions.

## 2 Materials and methods

### 2.1 Experimental facility

The study was conducted in environment-controlled plant growth chambers at the Soil-Plant-Atmospheric-Research (SPAR) facility, Environmental Plant Physiology Laboratory, Mississippi State University, Mississippi, USA, during the summer of 2023. The SPAR chambers precisely monitor and control the set CO_2_ and temperature through an automated control system. Each SPAR chamber consists of a steel soil bin (1 m in height × 0.5 m in width) along with a clear, transparent Plexiglas acrylic chamber (2.5 m in height × 1.5 m in width), designed to house the plant canopy. Each SPAR unit is equipped with a heating and cooling system to control the temperature. These SPAR units were sealed to enable gas exchange monitoring but included a door for easy access to take measurements when necessary. The Plexiglas chambers are 1.27 cm thick and permit 97% visible solar radiation. The chambers were in the open sunlight; therefore, the plants grew under natural sunlight conditions. The details and specifications of the SPAR units have been presented by Reddy et al. (2001).

### 2.2 Experimental setup and treatments

An Upland cotton cultivar, DP 1646 B2 XF, a mid-full-season cultivar accounting for 21.35% of total US cotton acreage in 2020, was used in the study. The cultivar is characterized by triple-stacked herbicide tolerance (dicamba, glyphosate, and glufosinate) and insect control (Bollgard II technology), known as Bollgard II XtendFlex technology. Ninety-six pots (15 cm diameter × 65 cm height) filled with fine sand and topsoil mix in a 3:1 volume ratio (87% sand, 2% clay, and 11% silt) were used for growing the plants. Twelve pots were placed in each SPAR unit. The pots consisted of a hole of 1 cm diameter at the bottom and were filled with 250 g of gravel to facilitate easy drainage. Four seeds were sown in each pot and thinned to one plant per pot after the fourth leaf stage. The plants were grown under ambient outdoor conditions up to flowering. The water and nutrients were supplied through Hoagland’s nutrient solution (Hewit, 1953) thrice a day. The irrigation volume was adjusted according to the daily evapotranspiration (Reddy et al., 2001).

At flowering (60 days after planting), the plants were transferred to the SPAR chambers, which had been preconditioned to the specific temperature and CO_2_ concentrations. The treatments include four-day/night temperature conditions of optimum (OT, 33/21°C), high nighttime (OT+HNT, 33/24°C), high temperature (HT, 36/24°C), and high temperature with increased nighttime temperature (HT+HNT, 36/28°C) at two CO_2_ concentrations of 425 ppm (aCO_2_) and 725 ppm (eCO_2_). The experiment was conducted in a completely randomized design with 12 replications per treatment. The selection of the optimum temperature was based on the average summer temperature in Mississippi. Daytime temperatures were regulated from sunrise to sunset, while nighttime temperatures gradually changed over 30 minutes after sunset. Black netted shade cloths were installed around the edges of the Plexiglas chambers to mimic the effect of border plants. The shade netting was periodically adjusted according to plant growth.

### 2.3 Trait measurements

#### 2.3.1 Plant growth traits

The plant height and number of mainstem nodes were measured during the harvest stage. The plants were harvested 135 days after sowing, which was 75 days after the treatment. The plant height was measured using a ruler from the cotyledonary node to the stem apex. The number of mainstem nodes (plant^-1^) was counted, eliminating the cotyledonary node up to the uppermost node with a fully unfolded leaf.

#### 2.3.2 Boll production and retention

The bolls produced in each treatment were tagged daily with the date of flowering throughout the experiment for about 68 days (60–128 days after planting). The abscised bolls were collected and counted daily from each experimental SPAR unit, with the collection date recorded. At the end of the experiment, the bolls were monitored daily for their opening, which was indicated by the cracking of the boll and the appearance of lint. The boll opening date was marked on the tag beside the boll production date. This resulted in attaining the boll maturation period (BMP, days) as the time taken from the day of anthesis to the day of boll dehiscence. At the time of final harvest, 135 days after planting, the number of fruiting sites and bolls produced and retained were counted on all the plants. The boll retention (%) was accounted for by dividing the number of retained bolls at harvest by the total number of bolls produced per plant in each treatment.

#### 2.3.3 Pollen and ovule production

The flowers were collected between 18.00 and 19.00 h before anthesis. The number of anthers per flower, ovules per boll, and pollen grains per anther were counted in each experiment in three replications. The pollen grains were counted using a Stereo microscope (Nikon SMZ800, Kanagawa, Japan), at 10X magnification by cutting the indehisced anther with a needle in a drop of water.

The pollen grains were collected from the newly opened flowers, to study their morphology. The anthers were tapped onto Petri plates to collect pollen grains from four different plants in each treatment. The pollen grains were then bulked to mount on a stub for scanning. The indehiscent anthers were burst open using a needle, and the pollen grains were collected into a Petri dish. The pollen grains were mounted onto a steel stub, and it was coated with platinum in an EMS150T ES sputter coater (Quorum Technologies, Electron Microscopy Sciences, PA, USA). The samples were viewed under a scanning electron microscope (JEOL JSM-6500F, JEOL USA, Inc., MA, USA) at an accelerating voltage of 5 kV. The images were taken at a magnification of 400.

#### 2.3.4 Seed cotton and lint yield

The plants were harvested after 135 days of sowing. The opened bolls were collected at the time of harvest. Bolls were separated into burr, lint, and seed, and then weighed to obtain the seed cotton weight (g), lint weight (g), and lint (%). Lint yield was determined after ginning the seed cotton using a roller gin. Lint from each treatment was pooled across twelve plants, and three sub-samples of 15-20 g from each treatment were subjected to fiber quality assessment using High Volume Instrumentation (HVI) by the Fiber and Biopolymer Research Institute at Texas Tech University, Lubbock, TX, as described by (Davidonis and Hinojosa, 1994). The analysis provides the fiber length (mm), fiber strength (g tex^-1^), micronaire (unitless), and uniformity (%).

### 2.4 Data analysis

The analysis of variance was performed using a two-way factorial completely randomized design in R Studio using doebioresearch (Popat and Banakara, 2020). The *Post-hoc* tests were conducted to identify treatment differences at a 0.05 significance level using an LSD test. Graphical representations of the outcomes were generated using SigmaPlot 13.0 (Systat Software Inc., San Jose, CA, USA) and GraphPad Prism 8.00 (GraphPad Software, San Diego, CA, USA).

## 3 Results

The cotton cultivar exhibited significant responses to temperature differences during flowering and boll development periods. All measured parameters were significantly different (p < 0.001 and p < 0.05) due to temperature variations, except for fiber uniformity and fiber strength (**Table 1**). However, the eCO_2_ during the cotton reproductive stage only altered the boll maturation period (p <0.001). The flower characteristics and fiber micronaire were influenced by the interaction of temperature and CO_2_ treatments (p < 0.01 to p < 0.05).

**Table 1:** Significance levels of impact of ambient (aCO_2_, 425 ppm) and elevated (eCO_2_, 725 ppm) CO_2_ levels under varying day/night air temperatures: optimum (OT: 33/21°C), high nighttime (OT+HNT: 33/24°C), high temperature (HT: 36/24°C), and high day and nighttime (HT+HNT: 36/28°C) in controlled Soil-Plant Atmospheric Research (SPAR) chambers on growth, reproductive, and fiber quality parameters of cotton.

Parameters	Temperature	CO_2_	Temperature × CO_2_
Plant height (cm)	1.16e-09 ***	0.425	0.537
Nodes (no. plant^-1^)	2.14e-10 ***	0.709	0.524
Bolls produced (no. plant^-1^)	<2e-16 ***	0.149	0.175
Boll maturation period (days)	< 2.2e-16 ***	0.0003 ***	0.329
Anthers (no. flower^-1^)	2.97e-6 ***	0.564	0.003 **
Ovules (no. boll^-1^)	3.22e-6 ***	0.425	0.008 **
Pollen grains (no. anther^-1^)	6.25e-13 ***	0.949	0.007 **
Micronaire (unitless)	0.024 *	0.522	0.007 **
Fiber length (mm)	0.0001***	0.314	0.525
Uniformity (%)	0.067	0.343	0.111
Fiber strength (g tex^-1^)	0.403	0.935	0.314

Values indicate the p-value at the 0.05 level of significance. *, **, and *** represent statistical significance at p <0.05, 0.01, and 0.001, respectively. p-values above 0.05 are non-significant.

### 3.1 Effect of day/night temperature differences on plant height and node number

**Figure 1:**
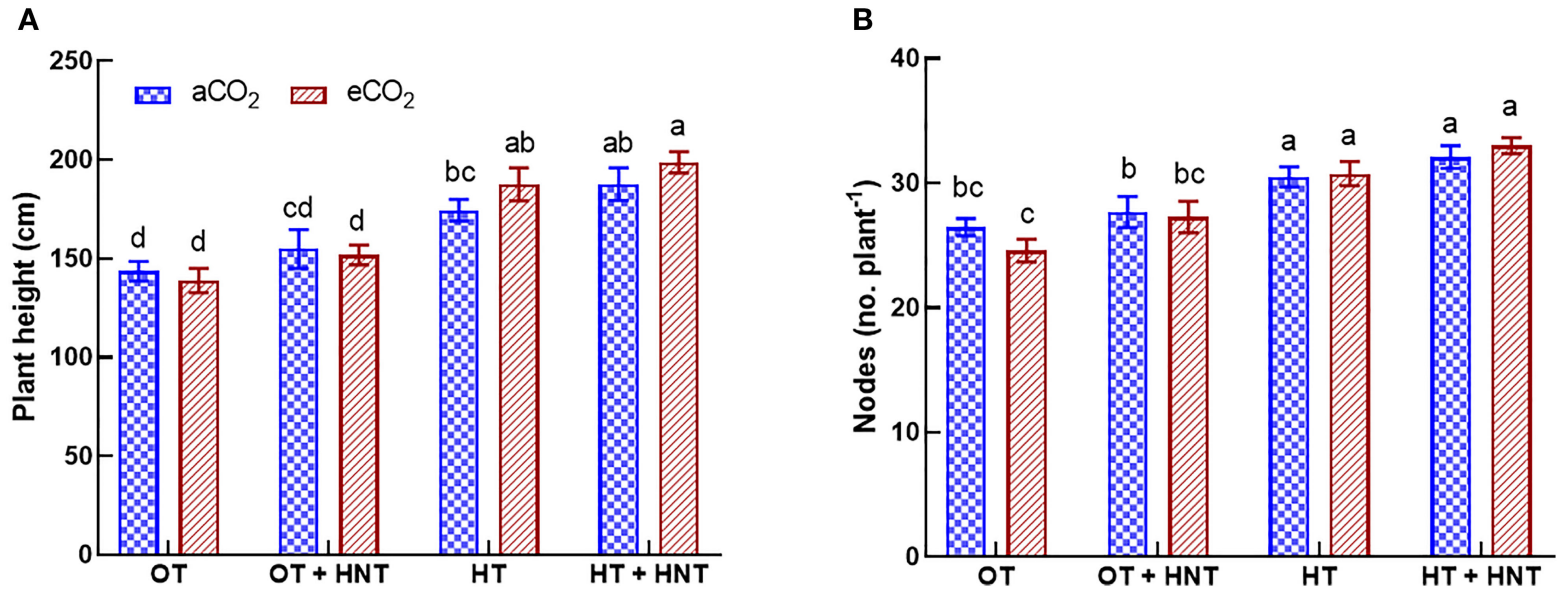
Day/Night air temperatures (OT: 33/21°C, OT+HNT: 33/24°C, HT: 36/24°C, HT+HNT: 36/28°C) and CO2 (aCO2: 425 ppm, eCO_2_: 725 ppm) effects on **(A)** plant height and **(B)** number of nodes (plant^-1^) during cotton boll development stage. The bars are the treatment mean ± SE of twelve replications. The letter above the bar indicates significance at p< 0.05. The treatments with the same letters are non-significant.

The plants grown under HT and HT+HNT had higher plant heights compared to those under OT and OT+HNT (**Figure 1A**). However, plant height did not show a positive response to eCO_2_ in any of the temperature treatments. On average, plant height increased by 6% under OT+HNT compared to OT across both CO_2_ conditions. However, the difference was statistically nonsignificant. At the same time, a significantly higher increase of 28 and 37% was observed under HT and HT+HNT conditions, respectively, compared to OT. A similar observation was made for the number of nodes under various temperature treatments studied. The number of nodes under eCO_2_ was comparable to that under aCO_2_ in all temperature conditions (**Figure 1B**). In contrast, plants grown under HT and HT+HNT conditions produced 31 and 33 nodes per plant, respectively, compared to 26 under OT, representing 20% and 27.5% more nodes, respectively. At the same time, the plants under OT+HNT treatment had 27 nodes, which were on par with OT.

### 3.2 Effect of day/night temperature differences on boll production and retention

**Figure 2:**
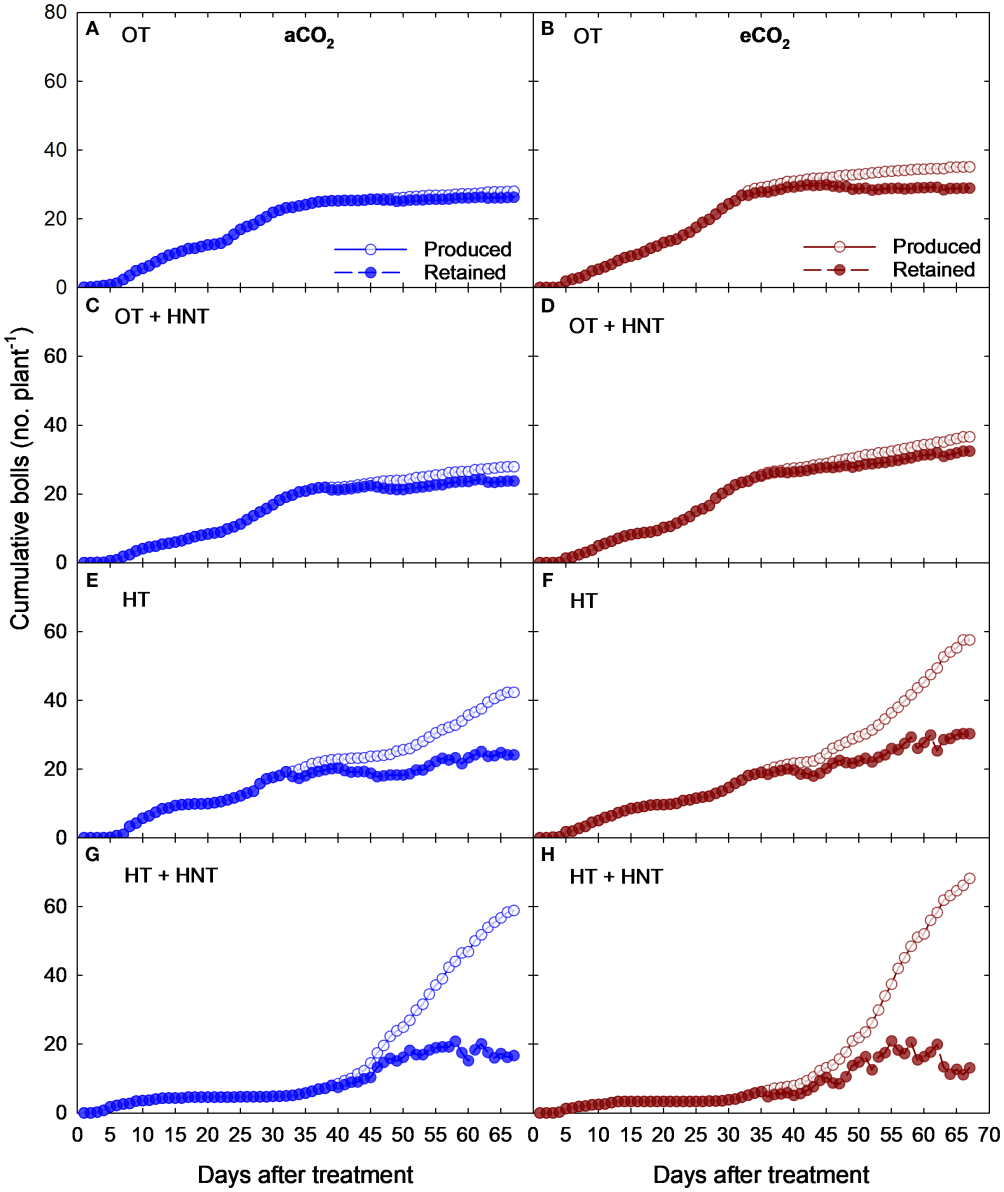
Cumulative number of boll production and retention (no. plant^-1^) at aCO_2_ (425 ppm) and eCO_2_ (725 ppm) concentrations under day/night temperatures of **(A, B)** OT (33/21°C), **(C, D)** OT + HNT (33/24°C), **(E, F)** HT (36/24°C), and **(G, H)** HT + HNT (36/28°C) in cotton. The X-axis indicates the days after treatment (0–68 days), which is equivalent to 60–128 days after sowing. The experiment consisted of 12 plants per square meter.

The cumulative number of cotton bolls produced and retained varied across CO_2_ concentrations and temperature treatments (**Figure 2**). The following results of boll production and retention are expressed on a per-plant basis. Plants grown under OT produced bolls at a constant rate from 7 days after treatment (DAT) to 36 DAT, incorporating an average of 0.6 bolls per day at aCO_2_ (**Figure 2A**), reaching a maximum of 25 bolls at 36 DAT. After 36 DAT, the plants ceased their boll production, incorporating only 3 additional bolls by the end of the experiment. The plants retained nearly all the bolls produced under OT and aCO_2_, showcasing a similar cumulative boll production curve. The boll production under OT and eCO_2_ conditions followed a similar pattern with a slight increase in boll production and retention (**Figure 2B**). The boll addition rate was static up to 38 days with an average production of 0.9 bolls per day. In the same way as OT, the plants exposed to OT+HNT produced bolls with minimal boll abscission under aCO_2_ and eCO_2_ conditions with a slightly higher number compared to OT (**Figures 2C, D**). In the initial days of OT+HNT treatment from 6 to 35 DAT, the plants produced an average of 0.7 and 0.8 bolls per day under aCO_2_ and eCO_2_ conditions, respectively. By the end of the experiment, the total number of bolls produced and retained were 28 and 23 under aCO_2_ and 36 and 30 under eCO_2_, respectively. However, when the plants are exposed to HT and HT+HNT conditions, the boll production increased towards the end of the experiment (**Figures 2E-H**). Plants grown under HT and eCO_2_ produced more bolls, reaching 57 bolls at 68 DAT compared to 43 bolls under aCO_2_. However, at the time of harvest, the number of retained bolls was 14 and 15 under aCO_2_ and eCO_2_, respectively. The plants exposed to HT+HNT conditions produced approximately 13 bolls by 35 DAT with 100% retention. While a sharp increase in boll production was observed after being exposed to HT+HNT conditions beyond 45 days, producing 2–4 flowers a day. However, the majority of the bolls abscised as the days progressed towards the end of the experiment, retaining only 10 bolls by 68 DAT, compared to 60 and 80 bolls produced under aCO_2_ and eCO_2_ conditions, respectively.

**Figure 3:**
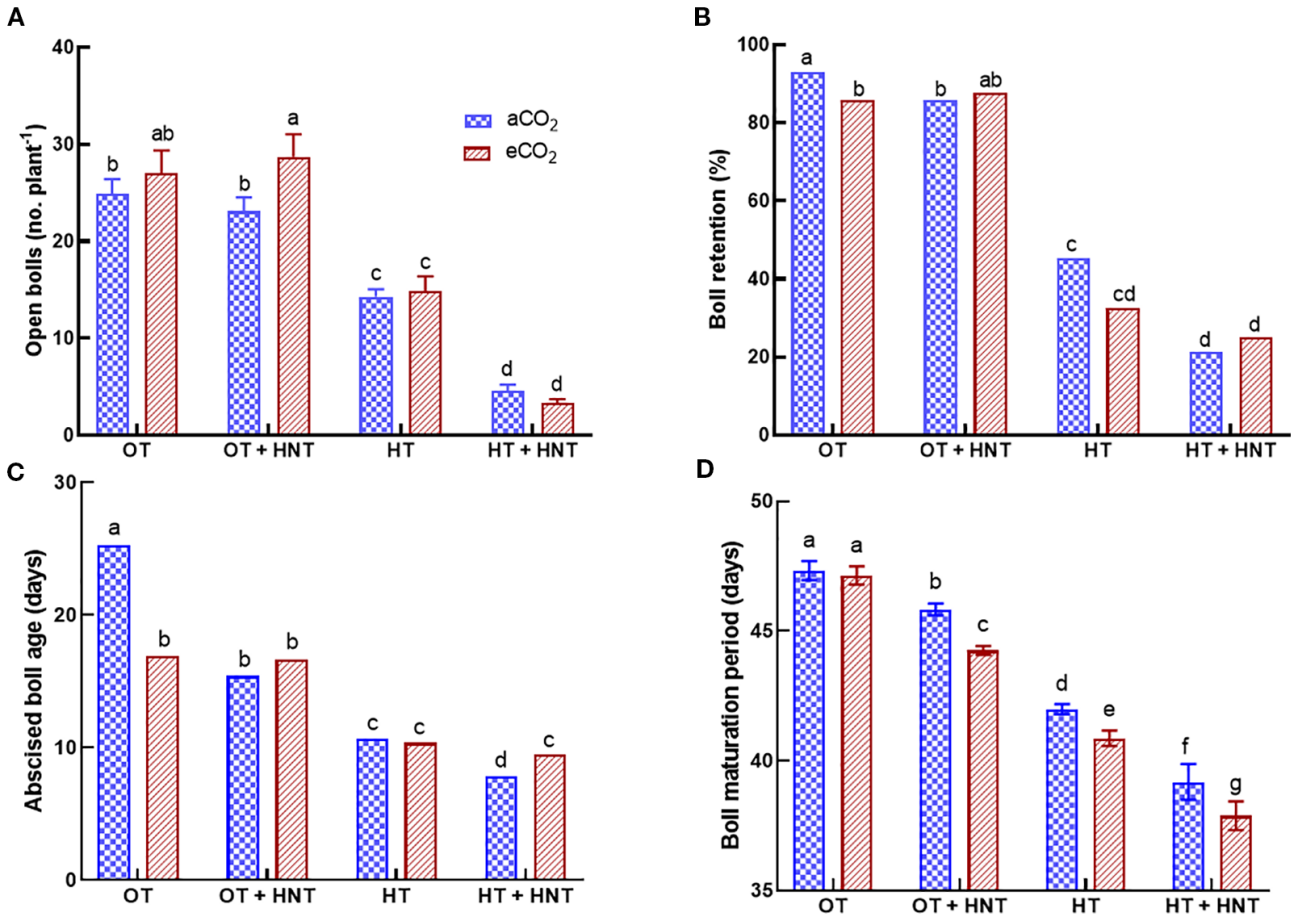
Day/Night air temperatures (OT: 33/21°C, OT+HNT: 33/24°C, HT: 36/24°C, HT+HNT: 36/28°C) and CO2 (aCO2: 425 ppm, eCO_2_: 725 ppm) effect on **(A)** number of open bolls, **(B)** boll retention (%), **(C)** abscised boll age (days), and **(D)** boll maturation period (days) on cotton. The bars are the treatment mean ± SE of 12 replications. The letter above the bar indicates statistical significance at p< 0.05. The treatments with the same letters are non-significant.

The flower tagging was terminated at 68 DAT. In contrast, the collection of abscised bolls continued until harvest. At the time of harvest, the bolls produced after 70 DAT were recorded and incorporated into the boll retention (%). The total number of open bolls significantly reduced under HT and HT+HNT conditions (**Figure 3A**). Under aCO_2_ conditions, the total number of open bolls reduced by 44% and 82% under HT and HT+HNT conditions compared to OT. Similarly, under eCO_2_, the reductions were 44% and 88% under HT and HT+HNT conditions, respectively, compared to OT. After the harvest, the boll retention (%) was estimated using the total number of bolls produced and retained at the time of harvest. The plants grown under OT retained 93% of the produced bolls under aCO_2_, while under eCO_2,_ the retention rate decreased to 86% (**Figure 3B**). However, under OT+HNT conditions, the boll retention was 86% and 88% under aCO_2_ and eCO_2_ conditions. On the other hand, over half of the produced bolls were abscised under HT conditions under both aCO_2_ (45%) and eCO_2_ (32%) conditions. While the plants grown under HT+HNT retained only 21 and 25% bolls under aCO_2_ and eCO_2_ conditions. A reduction in boll retention (%) was observed with an increase in average temperature.

The days between boll production and the abscised boll collection date were used to estimate the age of the abscised bolls. The average age of abscised bolls produced under OT was 25 days under aCO_2_, whereas it decreased to 17 days under eCO_2_ (**Figure 3C**). Under OT+HNT conditions, the decrease was 39% and 34% under aCO_2_ and eCO_2_, respectively, compared to OT and aCO_2_. With the increase in temperature, the plants abscised their bolls quickly. Under HT, the bolls abscised within 10 days of production under both CO_2_ conditions, resulting in a 58% reduction compared to OT. While under HT+HNT conditions, the abscission occurred at 8 and 9.5 days under aCO_2_ and eCO_2_, respectively.

The BMP (days) decreased with an increase in average temperature (**Figure 3D**). The average BMP of bolls produced under OT was 47 days, regardless of the CO_2_ conditions. When the plants were exposed to OT+HNT, the days decreased to 46 (aCO_2_) and 44 (eCO_2_) days. Those exposed to OT+HNT also showed a significant decline in BMP under eCO_2_ compared to aCO_2_. Under HT and HT+HNT conditions, the BMP was further reduced to 42 and 39 under aCO_2_, respectively, while it was 41 and 38 under eCO_2_, respectively. This accounted for a decline of 12% and 18% in the number of days to BMP under HT and HT+HNT conditions, respectively, compared to OT. The outcome shows that eCO_2_ significantly reduces the BMP under higher temperature conditions beyond the optimal temperature (OT).

### 3.3 Effect of day/night temperature differences on ovule and pollen production

**Figure 4:**
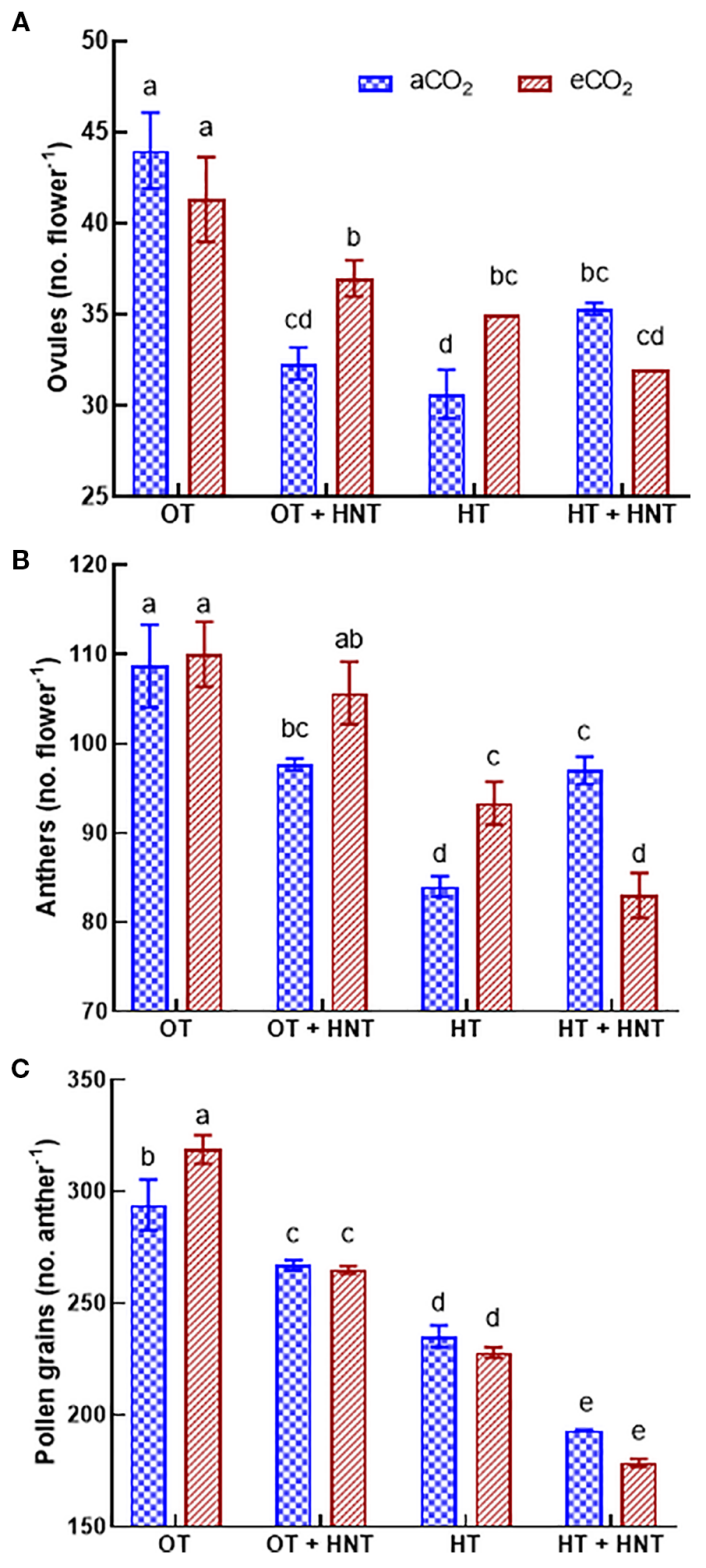
Day/Night air temperatures (OT: 33/21°C, OT+HNT: 33/24°C, HT: 36/24°C, HT+HNT: 36/28°C) and CO2 (aCO2: 425 ppm, eCO_2_: 725 ppm) effect on **(A)** ovules (no. boll^-1^), **(B)** anthers (no. flower^-1^), and **(C)** pollen grains (no. anther^-1^) of cotton. The bars are mean ± SE of 3 replications. The letter above the bar indicates significance at p< 0.05. The treatments with the same letters are non-significant.

The number of ovules decreased under OT+HNT, HT, and HT+HNT conditions compared to OT under both CO_2_ environments (**Figure 4A**). The number of ovules in flowers produced under OT+HNT was reduced by 26% compared to OT at aCO_2_. In comparison, the reduction was 16% under eCO_2_ and OT+HNT conditions. Similarly, under HT, the number of ovules decreased by 30 and 20% under aCO_2_ and eCO_2_ conditions, respectively, compared to OT. On the other hand, in HT+HNT conditions, eCO_2_ did not result in any significant gain in ovule number, resulting in an average decline of 21% compared to OT. Under aCO_2_, flowers produced under HT had the highest reduction in number of anthers by 23%, followed by OT+HNT and HT+HNT conditions, each with 11% reduction compared to OT (**Figure 4B**). While under eCO_2_, OT+HNT had a comparable number of anthers with OT, while under HT and HT+HNT conditions, the number reduced by 14 and 24, respectively, compared to OT. An 8% increase in pollen number per anther was observed under eCO_2_ conditions at OT compared to aCO_2_ (**Figure 4C**). However, at different high night and day temperature conditions, the eCO_2_ did not influence the pollen number. The flowers produced under OT+HNT, HT, and HT+HNT conditions had 13%, 24%, and 39% reductions in pollen number, respectively, compared to OT.

**Figure 5:**
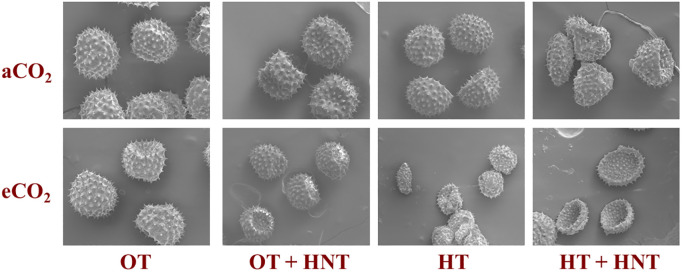
Day/Night air temperatures (OT: 33/21°C, OT+HNT: 33/24°C, HT: 36/24°C, HT+HNT: 36/28°C) and CO2 (aCO2: 425 ppm, eCO_2_: 725 ppm) effects on cotton pollen morphology. The figure is magnified at 10 µm.

Scanning electron microscopic images of pollen grains produced in plants grown under OT+HNT, HT, and HT+HNT showed morphological differences compared to pollen produced under OT (**Figure 5**). Pollen grains appeared round and uniform with spiny exine under OT. When plants were exposed to OT+HNT, pollen grains remained largely intact, with slight abnormalities in shape, indicating minor stress-induced changes. The abnormalities were more evident when the growing temperature increased to HT and HT+HNT conditions. The pollen grains produced under HT exhibit signs of shrinkage, deformation, and irregular shapes. The pollen grains under HT+HNT exhibited the most severe impact on morphology. Many of the pollen grains appeared shriveled, collapsed, or fragmented, indicating compromised viability. The eCO_2_ conditions during pollen development did not alleviate the high-temperature stress damage; in fact, under HT+HNT, the degradation was higher under eCO_2_ conditions. The anther indehiscence observed under HT and HT+HNT temperature conditions was primarily due to the reduced pollen volume and number.

### 3.4 Effect of day/night temperature differences on seed cotton and lint yield

**Figure 6:**
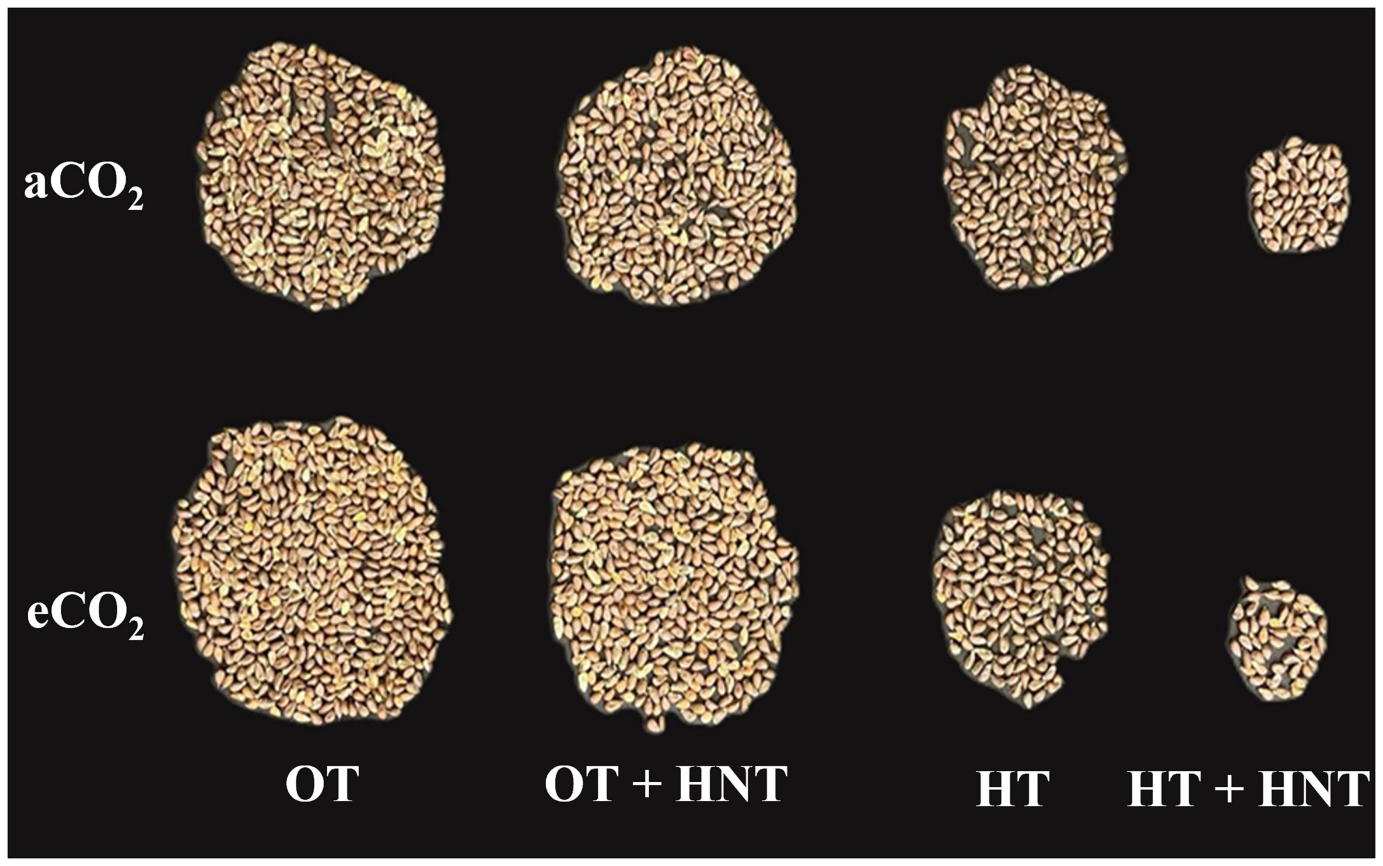
Seed yield under different Day/Night air temperatures (OT: 33/21°C, OT+HNT: 33/24°C, HT: 36/24°C, HT+HNT: 36/28°C) and CO2 (aCO2: 425 ppm, eCO_2_: 725 ppm) treatments in cotton. Image represents 50% yield of a plant.

The seed and lint yield of cotton was significantly influenced by the day and night temperature alterations in the growing environment (p < 0.001) (**Figure 6**; **Table 2**). While the eCO_2_ did not influence the yield parameters under either temperature condition. On the other hand, the interactions between temperature and CO_2_ influenced all the yield parameters (p < 0.05 to 0.001), except for lint percentage.

**Table 2:** Different diurnal air temperatures (optimum (OT): 33/21°C, high temperature (HT): 36/24°C, high nighttime (OT+HNT): 33/24°C, and high day and nighttime (HT+HNT): 36/28°C) and CO_2_ (aCO_2_: 425 ppm; eCO_2_: 725 ppm) effects on cotton seed and lint yields in controlled Soil-Plant Atmospheric Research (SPAR) chambers.

Temperature	CO_2_	Seed cotton weight (g)	Lint weight(g)	Seed weight (g)	Lint (%)	100 seed weight (g)
OT (33/21 °C)	aCO_2_	94.77bc	38.48b	51.48b	42.39a	8.01c
	eCO_2_	114.88a	48.24a	66.42a	44.26a	8.08c
OT + HNT (33/24 °C)	aCO_2_	79.44c	31.65c	43.85c	41.06a	7.39de
	eCO_2_	99.69ab	35.34bc	51.03b	39.31a	7.55d
HT (36/24 °C)	aCO_2_	50.67d	19.38d	31.31d	39.48a	8.26b
	eCO_2_	43.92d	13.13e	27.62d	31.72c	8.54a
HT + HNT (36/28 °C)	aCO_2_	14.35e	4.72f	9.59e	31.51c	7.51d
	eCO_2_	9.55e	2.91f	6.75e	32.30c	7.28e
p-value	Temperature	< 2e-16 ***	< 2.2e-16 ***	< 2.2e-16 ***	7.8e-05 ***	5.07e-15 ***
	CO_2_	0.13	0.37	0.07	0.32	0.11
	Temperature × CO_2_	0.04 *	0.0002***	0.003 **	0.20	0.002 **

*, **, and *** represent statistical significance at p <0.05, 0.01, and 0.001, respectively. The letter near the values indicates significance at p< 0.05. The treatments with the same letters are non-significant.

Seed cotton weight was reduced by 16% and 13% under OT+HNT conditions in aCO_2_ and eCO_2_ environments, respectively, compared to OT under the corresponding CO_2_ conditions. However, these differences were comparable to those of OT. Under both OT and OT+HNT conditions, seed cotton weight increased by 21% and 25%, respectively, due to eCO_2_ levels compared to aCO_2_. When the plants were exposed to HT conditions, the seed cotton weight declined sharply by 47%, reaching 50.7 g compared to 95 g under OT at aCO_2_. While under eCO_2,_ the reduction was 62% compared to OT. The most severe reduction in seed cotton weight was observed under HT+HNT, at 85% and 92% under aCO_2_ and eCO_2_ conditions, respectively, compared to the corresponding CO_2_ environments under OT. Under high temperatures, the effect of eCO_2_ was non-significant.

The lint and seed weights were higher in plants grown under OT at eCO_2_ conditions by 25% and 29%, respectively, compared to those grown under aCO_2_. However, the CO_2_ did not influence temperature effects, except for OT+HNT, which affected seed weight, and HT, which affected lint weight. A reduction of 18, 50, and 88% lint weight was observed in plants grown under OT+HNT, HT, and HT+HNT, respectively, compared to OT under aCO_2_. Although the lint weight did not differ significantly between CO_2_ conditions under OT+HNT, the seed weight showed a 16% increase under eCO_2_ compared to aCO_2_. The average seed weight under HT and HT+HNT was 29.5 and 8.17 g, respectively, representing approximately 43% and 84% reductions compared to OT under aCO_2_. The lint percentage remained relatively stable under OT and OT+HNT, with values ranging from 39% to 44%, showing no significant decline under eCO_2_. However, a notable reduction was observed under HT and HT+HNT, with lint percentages dropping to 31.72% and 32.30%, respectively, under eCO_2_. The 100-seed weight was significantly reduced under both high night temperature treatments, including OT+HNT and HT+HNT. Under OT+HNT, the 100-seed weight declined to 7.47 g compared to 8.05 g observed under OT conditions. A similar reduction was noted under HT+HNT, with a recorded weight of 7.39 g. However, the 100 seed weight increased under HT conditions (8.26 g), which was further enhanced under the eCO_2_ conditions, reaching 8.54 g.

### 3.5 Effect of day/night temperature differences on fiber quality

**Figure 7:**
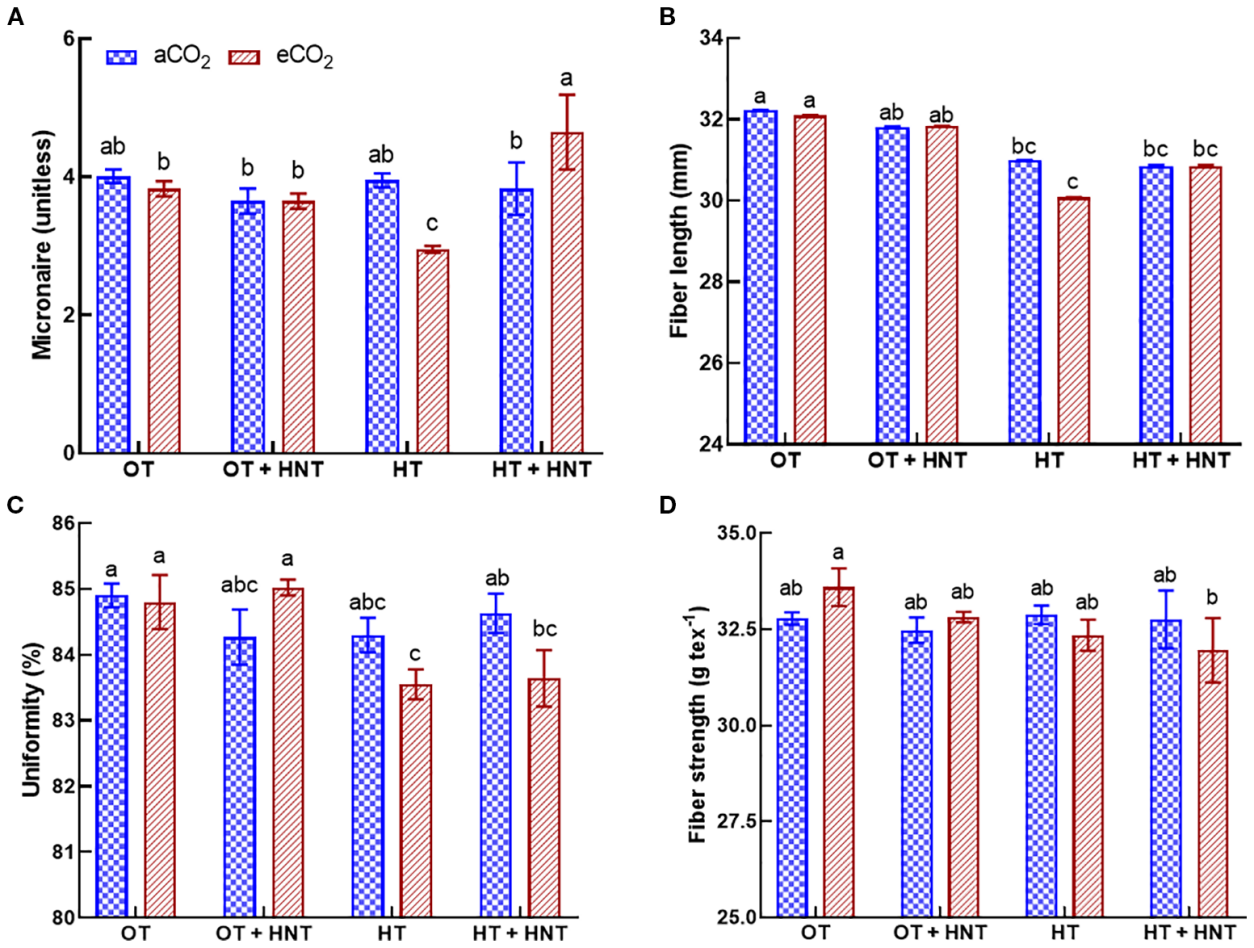
Day/Night air temperatures (OT: 33/21°C, OT+HNT: 33/24°C, HT: 36/24°C, HT+HNT: 36/28°C) and CO2 (aCO2: 425 ppm, eCO_2_: 725 ppm) effect on **(A)** micronaire, **(B)** fiber length, **(C)** uniformity, and **(D)** fiber strength of cotton lint. Bars represent the treatment mean ± SE of 3 replications. The letters above the bars indicate a statistically significant difference at p < 0.05. The treatments with the same letters are non-significant.

The cotton fiber produced under different temperature conditions only influenced the fiber micronaire and fiber length (**Figures 7A, B**). The fiber strength and uniformity were not affected by the change in growing temperature or CO_2_ conditions (**Figures 7C, D**). The lowest micronaire number was observed under HT and eCO_2_ environments, with a reduction of 25% compared to OT and aCO_2_ (**Figure 7A**). Similarly, the fiber produced under HT had the lowest fiber length, with a decline of 7% compared to OT and aCO_2_ (**Figure 7B**).

## 4 Discussion

The study highlights the decline in reproductive performance and yield potential of cotton plants grown under increased daytime and/or nighttime temperatures in both ambient and elevated CO_2_ environments. The study specifically focuses on the boll production and retention efficiency of cotton when exposed to temperature fluctuations during the reproductive stage. It reports the temporal changes in boll production during the crop season. We observed a drastic increase in boll production under HT and HT+HNT conditions towards the end of the season, with approximately 50 bolls produced under HT and 70 bolls under HT+HNT conditions, averaged across the CO_2_ environments. However, the produced bolls were affected by temperature, resulting in boll abscission. Significant changes in plant growth, flower characteristics, and seed yield were recorded in the study.

The plant height of cotton is a geometric trait that contributes to the biomass and harvest index (Jiang et al., 2016). The height of plants grown under HT and HT+HNT conditions increased by 28 and 37%, respectively, compared to OT, reaching almost 2 meters tall at HT+HNT and eCO_2_ conditions. In our study, we observed an increase in flower abscission towards the end of the experiment, resulting in greater availability of carbon sources in the plant under high temperatures. Temperatures damaging to fruit production may alter the balance between vegetative and reproductive growth, leading to increased growth of both the main stem and branches (Hatfield and Prueger, 2015; Allen et al., 2018). Cotton, being plastic in nature with an indeterminate growth habit, it translocated the photosynthates to the growing meristematic cells, adding more nodes and internodes, leading to increased plant height (Emara and El-Sayed, 2021; Naveed et al., 2023). This is manifested by an increased number of nodes observed under HT and HT+HNT conditions in both CO_2_ environments, resulting in 33 nodes under HT+HNT compared to 26 nodes under OT. It is also important to note that the plants under eCO_2_ had slightly higher plant height and a non-significant increase in the number of nodes, which is attributed to the increased rate of photosynthesis under CO_2_ fertilization (Zhang et al., 2017; Wang et al., 2020; Gong et al., 2025). These changes in plant height and nodes contributed to an increased number of leaves and a higher leaf area index (Li et al., 2015), which in turn contributed to a higher rate of light interception and a subsequent increase in carbon fixation. In turn, this contributes to the availability of a carbon source for further addition of nodes and internodes. The plant height and number of nodes grown under OT+HNT conditions were similar to those in plants under OT.

As the plant grows and adds more nodes, it contributes to the production of fruiting sites. This is evident from the increased production of bolls towards the end of the growing season under HT and HT+HNT conditions with less boll retention (**Figure 2**). The final yield of seed and lint depends on the number of bolls produced and their retention. The plants allocated more carbon sources towards the first-formed bolls up to 35 days after treatment, retaining all the developed bolls. Upon increased exposure to HT and HT+HNT conditions, the plants were unable to support the bolls, resulting in abscission. The increased availability of carbon source contributed to the increased production of bolls under eCO_2_ conditions in all the temperature environments (Reddy et al., 1995b; Jans et al., 2021; Gong et al., 2025). Under high temperatures, the number of fruiting sites on vegetative branches also increased, as observed in other studies (Reddy et al., 1995a; Abro et al., 2025). On average, the boll retention percent under normal conditions exceeds 80%, which is essential for achieving a good lint yield. However, under HT and HT+HNT conditions, most of the bolls failed to reach maturity and dropped within 7 to 10 days. As a result, boll retention was significantly reduced to only 28% under HT and 19% under HT+HNT conditions. The BMP is the time interval between flowering and boll opening. The study also observed a reduction in the BMP with an increase in temperature, whether during daytime or nighttime. The eCO_2_ further reduced the BMP compared to aCO_2_ at all temperature conditions above OT. Under increased temperatures (HT and HT+HNT) above the optimum, the plants attempt to shorten the life cycle of a boll to 40 days, compared to 47 days under optimal temperatures (OT). Even the BMP under OT+HNT was reduced by 2–3 days compared to OT.

The reduced seed and lint yield observed in the study can be attributed to multiple reasons. The number of male and female reproductive organs is a yield-determining factor as the number of seeds per boll depends on the number of ovules per locule, and successful fertilization depends on the pollen load and its quality (Song et al., 2015). The study observed a significant reduction in both crucial traits, including the number of anthers per flower (**Figure 4**). Reproductive development is susceptible to high temperatures, both before and after anthesis (Lokhande and Reddy, 2014; Song et al., 2015; Bista et al., 2025). The number of ovules flower^-1^ was reduced by 26% and 30% under OT+HNT and HT conditions, respectively, under aCO_2_, compared to OT. Increased carbon availability under eCO_2_ minimized the adverse effect of increased temperatures under OT+HNT and HT conditions, resulting in only 16 and 20% lower numbers of ovules, respectively. However, CO_2_ fertilization did not help mitigate the adverse effect of increased day and night temperatures on cotton. As discussed earlier, cotton plants under high temperatures divert photosynthate to vegetative biomass rather than to reproductive structures, thereby limiting available resources for producing ovules (Lokhande and Reddy, 2014; Snider and Adegbenro, 2025). A similar observation was made for several pollen grains anther^-1^, resulting in a reduction of 24% and 39% under HT and HT+HNT conditions, respectively, compared to OT. At OT, however, it did not enhance pollen production under increased temperatures above OT. The reduced availability of non-structural carbohydrates in the floral buds under high temperatures may have caused a reduction in male and female gametophyte development in cotton plants (Borghi et al., 2019).

The quality and integrity of pollen are crucial for successful fertilization of the ovule and thus the boll and seed set. Pollen development consists of two sequential stages: microsporogenesis and microgametogenesis, which are highly sensitive to temperature fluctuations (Masoomi-Aladizgeh et al., 2021; Lohani et al., 2025). The study observed significant structural alteration of pollen grains with increasing temperature, regardless of CO_2_ availability. The shrinkage and deformation observed at temperatures above OT indicate a reduced availability of potential pollen grains for fertilization, resulting in insufficient pollen germination and pollen tube growth (Song et al., 2014; Qian et al., 2025). It is independent of carbon availability and is mainly influenced by temperature conditions. The release of pollen grains from the anther, termed anther dehiscence, is critical for successful fertilization as it ensures the transfer of viable pollen to the stigma, facilitating pollination and subsequent fruit development (Deng et al., 2021). Both growing conditions and genetic regulations influence this process (Wilson et al., 2011; Zheng et al., 2019; Miglani et al., 2025). We observed anther indehiscence under HT and HT+HNT conditions at both aCO_2_ and eCO_2_, highlighting the impacts of temperatures on cotton flowering and fertilization. This indehiscence could be due to a reduced number of pollen grains per anther and distorted pollen structure, leading to insufficient pressure for anther opening. Alternatively, it may result from temperature-induced disruption in gene expression required for anther dehiscence (Sakata and Higashitani, 2008; Lohani et al., 2025). The anther indehiscence was not observed under OT+HNT conditions, suggesting that a slight increase in night-time temperature (33/24 °C) compared to 33/21 °C does not affect the genes responsible for anther dehiscence. Additionally, our findings indicate that a minimum of 265 pollen grains of anther^-1^ is sufficient for successful anther dehiscence. A positive correlation between anther sterility and high temperatures was reported 15 and 16 days prior to anthesis, indicating that microgametophyte development was susceptible to high temperatures immediately after meiosis of the microspore mother cells (Song et al., 2014). This may have resulted in the retention of early-formed flowers under high-temperature conditions.

The seed and lint yield in cotton is a function of boll production and retention. The study showed that the reduction in boll retention, either due to unsuccessful fertilization and/or limited translocate allocation, contributed to the decline in seed and lint yield in cotton. This is also exacerbated by the shortened BMP, which reduces the time required to incorporate sufficient biomass into the developing bolls (Lokhande and Reddy, 2014). The highest seed cotton, lint, and seed weights were observed under OT conditions at eCO_2_ (**Table 2**). This is attributed to enhanced boll production, number of retained bolls, and the number of pollen grains in anther^-1^ under CO_2_ fertilization, due to increased availability of photosynthates. The increasing temperatures beyond 33/24 °C resulted in a drastic reduction, causing a decline of almost 90%, 88%, and 84% in seed cotton, lint, and seed weights compared to OT across the CO_2_ treatments. The reproductive failure of flowers developed under high-temperature conditions, accompanied by limited biomass partitioning towards the bolls, has led to the observed results. It is also important to note that the lint yield of plants under OT+HNT was reduced by 18% compared to OT, indicating the sensitivity of lint development to HNT conditions. The increased respiration under HNT conditions and limited cellulose supply to the bolls resulted in reduced lint weight under these conditions (Xu et al., 2021; Yang et al., 2023). This also contributed to the reduced weight of seeds under OT+HNT and HT+HNT conditions.

Fiber is the main economic product of the cotton crop, and the market value depends on its quality. The slight reduction in fiber length under HT and HT+HNT conditions is possibly affected by the limited biomass allocation to the boll development (Lokhande and Reddy, 2014; Xu et al., 2017; Abro et al., 2025). At optimum temperatures, the cotton fiber elongates over 2000-3000-fold within approximately 20 days after anthesis (Ruan et al., 2005). High temperatures during this period adversely affect the fiber elongation, in turn, shortening the fiber length (Lokhande and Reddy, 2014; Ali et al., 2025). The micronaire is an indirect measure of fiber fitness and maturity. Premium-quality fiber requires a micronaire value between 3.8 and 4.9 (Bange et al., 2009). In the eCO_2_ environment, fibers produced under HT and HT+HNT conditions had micronaire values of 2.95 and 4.65, respectively. The micronaire value below 3.8 indicates fiber immaturity, while values above 4.9 suggest coarser fibers with undesirable spinning characteristics (Luo et al., 2016). The temperature and eCO_2_ had no notable effects on fiber uniformity and strength. These fiber properties are likely primarily determined by the plant’s genetics and are only minimally influenced by growing conditions.

## 5 Conclusion

This study highlights the adverse effects of high air temperatures on cotton reproduction and yield, both in ambient and elevated CO_2_ conditions. Although boll production increased later in the season in response to high temperatures, high rates of abscission led to poor retention and ultimately reduced seed and lint yields. The observed increase in plant height and node numbers suggests a shift in resource allocation from reproductive growth to vegetative growth. High daytime and nighttime temperatures significantly hindered pollen production, anther dehiscence, and ovule formation, resulting in decreased fertilization success. While eCO_2_ levels improved photosynthesis, they did not fully counteract the yield losses caused by reproductive failures due to high temperatures. Under these high temperature conditions, seed cotton, lint, and seed yields experienced a sharp decline. These findings emphasize cotton’s vulnerability to rising air temperatures and the limited ability of elevated CO_2_ to mitigate these adverse effects. These findings should be further validated under field conditions to gain a more comprehensive understanding of plant behavior under current and future CO_2_ and temperature conditions.

## Data Availability

The original contributions presented in the study are included in the article/supplementary material. Further inquiries can be directed to the corresponding author/s.
